# Report of the Asylum for Military Lunatics, at Fort Clarence, from Its Establishment in May 1819, to May 1823

**Published:** 1824-06

**Authors:** S. A. Schetky

**Affiliations:** Surgeon to the Forces, (in charge of the Asylum.)


ART. III.—*Report of the Asylum for Military Lunatics, at Fort Clarence, from its Establishment in May* 1819, *to May* 1823. By S. A. SCHETKY, Surgeon to the Forces, (in charge of the Asylum.)

THE lot of the insane soldier was formerly not distinct from that of the maniacal pauper in civil life, and was a mere illustration of that general neglect, which consigns him to the cell or to unwatched liberty, and chance of destruction. The soldier was either turned over to friends, who, after long estrangement from his society, were not likely to hail with kindred sympathies an insane guest; or to establishments of such scope as did not adequately provide for the accumulated numbers of such patients. The motives for withdrawing from the scorn, or from the pity, of their fellow-subjects those brave, but unfortunate men, on many of whom the ravages of climate, or the chances of war—its hostile edge, its vicissitudes and privations, or the venial habits engendered by those vicissitudes, had done their work on the distinguishing attribute of our nature,—were motives emanating from the best-directed principles of protection. Whatever obvious advantages, on the score of economical propriety, may have been consulted or fulfilled by the establishment and conduct of this institution, such portion of the public who have visited its quiet retreats have felt that its value was not merely commensurate with its expediency, but stood on a patriotic basis. To bind together the interests of this fallen portion of the military community, by what shreds of instinct or of habit still remain of the web intellect, by consigning them to the care of functionaries, whose reason, more favoured, has weathered the same perils under which those have sunk, carries in it so palpably laudable a solicitude, that strangers are not less struck with the object than with the facility of its attainment.

The choice of Fort Clarence for a military asylum was dictated by views of convenience or of necessity, of which I am not the depositary. It presented, in a considerable degree, the requisites of security in the dwellings and of an ample space for exercise in the *terre pleine;* advantages which might be, and have been, improved. Its topographical situation need not be described, although some points in its locality are interesting, as connected with some of the prime objects of every such institution. It stands on an eminence, is visited by the most healthy winds from a rich and cultivated country, and the views from it are exceedingly beautiful and picturesque. The Medway winding through woods and fertile banks, and bearing on its bosom the bulwarks of naval war, and by its sinuous track giving irregular and striking features to the town; the prominent and round castle, bridge, dock-yards, roads, &c. &c. —are points on which the eye of the melancholy convalescent may rest with relief, or which the confirmed visionary may innocently people with his own creations. No spot in the whole vicinity presents those objects under such favourable forms. A wall, projecting from the tower of the fort, encloses a quadrangular space, adapted for the exercise of the soldier patients; for the same purpose, some easy adaptation of the dry ditch renders it available. The tower itself, with some bomb-proofs attached to the covered way, was found to afford ten rooms, or wards, of various sizes, from that necessary for two, to that for twelve soldiers. An excellent kitchen is connected with this. A small fort, by the brink of the river, furnishes a very convenient wash-house; another, at the opposite extremity of the works of the fort, was made to accommodate the steward, and to contain his stores.

As the beneficent and liberal purpose was extended to the relief and seclusion of insane officers, the saving of whose feelings and character from exposure and from irritation was, perhaps, a still more interesting object, the means were sought, by adapting a bomb-proof room, or series of rooms, in the centre of the works, to the comfortable accommodation of fifteen patients. It remained to provide for the disposal of a medical superintendant and keeper: for this, buildings were erected in situations convenient for the inspection of the patients. The whole of the terre pleine was assigned for the exercise of the officers.

The various wards and apartments for the patients of both classes are warmed by means of heated air, conducted in tubes from a central stove.

The proportion of hospital servants allotted to the care of the patients was from the first as now: one orderly to every ten patients, one ward-master, one steward, one civil keeper, one barber, one nurse. The olderly man attached to the officers was allowed an helper, in consequence of the greater range of space allowed to these gentlemen, and consequent increased difficulty of watching them; and because of their requiring various attentions, not habitually expected by the soldiers.

On the 10th May, 1819, the Asylum, thus preparatively organized, was opened for the reception of thirty patients, from Sir Jonathan Miles's establishment at Hoxton, who were brought down by the civil keeper. The admissions, discharges, deaths, from that period to the present, may now take a tabular form; after which, as no radical change of the system of administration has taken place,—no innovation,—there is no further room for an historical narrative of events. I shall therefore merge the past history in the description of the present state of the establishment.

| *Description of Patients.* | *Total admitted since* 10*th May,* 1819, *to* 10*th May,* 1823. | *Discharged since* 10*th May,* 1819, *to* 10*th May,* 1823. | | | | | | | | Total | *Total died since* 10*th May,* 1919, *to* 10*th May,* 1823. | *Remained on the* 10*th May,* 1823. |
|---|---|---|---|---|---|---|---|---|---|---|---|---|
| | | *Cured* | *Recent cases* | *Old cases* | *Relieved* | *Old cases* | *Unrelieved* | *Old cases* | *Recent cases* | | | |
| Officers ...... | 27 | 7 | 6 | 1 | 1 | 1 | 5 | 3 | 2 | 13 | 3 | 11 |
| Privates ...... | 126 | 30 | 28 | 2 | 1 | 1 | 3 | 3 | 0 | 34 | 26 | 66 |
| Extra Patients | 8 | 3 | 2 | 1 | 0 | 0 | 0 | 0 | 0 | 3 | 2 | 3 |
| | Total | 40 | 36 | 4 | 2 | 2 | 8 | 6 | 2 | 50 | 31 | 80 |

Of the patients, whose gross numbers I have thus exhibited, many had been confined in various asylums in London, or had been more recently sent from their regiments, after ineffectual attempts to check the progress of mental dilapidation. They were thus never altogether recent cases; many of them were old men; many had probably derived the insane predisposition from their parents, and it had been developed into active disease since their entrance into the army. The foreign regiments in our service also supplied several of the cases; and every country in Europe (except Spain and Portugal,) has sent its representative: the " extravagant and erring spirit" speaks with every tongue, and deforms every national physiognomy.

On entering the quadrangle, strangers generally appear struck with the sight of a number of grotesque figures, pacing or striding about in all directions, and with all degrees of rapidity, crossing at all angles, without jostling or appearing conscious of each other's presence, with the head sunk on the breast, or raised in invocations to the sky. The marble stare of inane reverie marks the locked absorption of some, and the more sickening smile or grin of inane complacency the loose passiveness of other minds. The total collapse of intellect, the absence of all but mere consciousness, characterises the automatic fixity of the idiot; and the fiery declamation or howls of the maniac repel the approach of the stranger. On the word of command to "fall in" being given, the remaining instinct of military obedience arranges them, though somewhat slowly and uncouthly, into two ranks, and controls their march into military time and order, round and round the quadrangle, or in the outer ground or terre pleine, assisted by the clarinet of a mad musician.* An inspection of the rooms shows the utmost cleanliness: the beds are very neatly made (viz. folded) up; no bad smells attack the nostrils; nor are the persons of the patients squalid, nor their beards of more than two days' growth.

All domestic offices, as cleaning, scowering the platters, making the beds, bringing the rations from the steward's store, laying the tables for meals, carrying the foul linen to the wash-house, are done by the more convalescent patients themselves; the services of the more intelligent part of whom are in every possible instance rendered available, for the assistance of the more helpless inmates. The duty of the orderlies is to superintend the execution of their patients' tasks, and to cut up their meat in small portions, to be eaten with the fingers. This last duty of the orderlies is done in a small anti-room, separated merely by a palisading from each ward: there also the instruments of security are kept, strait waistcoat, waist-belt with manacles attached, hobbles for the ankles, webs of girth, the wooden wedge and iron jug, or boat, for the forcible administration of food or medicine in refractory cases; and dishes and platters. Here also the respective orderlies sleep, in immediate presence of their charge.

The officers range over an ample space of sod, and enjoy the prospect, read magazines and newspapers, or novels, or classical authors; smoke; dose in the shade, or gaze on the sky; play at quoits, or skittles, or backgammon, watched by two orderlies. Their persons are not neglected; their individual peculiarities are sometimes striking and repulsive. Their dwelling consists of two sitting rooms and three bed rooms, furnished with very comfortable and neat beds, &c. A space is pallisaded off, to contain their spare clothing and moveables, to which, in most cases, they are not allowed access. A kind of kitchen,

* The uniformity of their dress, a loose flowing robe of flannel, and their general solemnity, give them something of a spectral appearance.

not used as such, contains the dishes, knives and forks, &c. &c. under the charge of the orderly. The officers eat with knives and forks, under close observation. Such as possess any accomplishment, as music or drawing, are encouraged to cultivate it; and some have done so, in a trivial desultory manner.

The buildings, not having been originally destined for their purpose, do not so completely unite the provisions for security with scope for exercise, as to supersede the necessity of constant vigilance. The outer walls are easily surmounted, and the fence of the terre pleine still more easily; the guard at the gates, changed every day, and not knowing the persons of the officer patients, might easily be deceived; the means of suicide, by a plunge into the ditch, are easily at hand; and none of these weak points can be debarred from the approach of the officers, without much inconvenient or injudicious restrictions. The only safeguard is the number and vigilance of the attendants: their prying eye must detect the nascent purpose, and give warning of the coming paroxysm. For this purpose the medical superintendant is assisted by a civil keeper, a person brought up to this peculiar profession, whose duty it is to range over every part of the establishment, and, when necessary, to seize and coerce, with the courage, and coolness, and gentleness, of accustomed power and address, such patients as may exhibit symptoms of rising ferocity: and these qualities are invariably observed in the exercise of such restrictive offices. Passion or vindictive feeling, it may safely be said, are banished from the breasts of the sane part of the family.

As connected with the welfare of every insane establishment, the object of classification is understood to hold an important place. It cannot, however, be cultivated to any considerable extent in Fort Clarence, from the want of spare room; every habitable part of the fort being now completely occupied. The most that can be done is, by frequent shifting of individual patients, to attain successively as many points of expediency, both in behalf of the patient's comfort, and for the convenience of superintendance. A gradual scale of promotion of the convalescents to a particular division of the asylum, according to their progressive improvement, has been adhered to as much as possible. Such patients, also, who are affected with paralysis and epilepsy, are especially placed together in a particular division, with a double allowance of aid from the servants. Such arrangements, however, are often traversed by occasional considerations. Thus, it has often been found necessary to place an improved patient among the most helpless class, for the sake of the benefit of his services; a compliment, of which many of the convalescents are highly sensible; and a patient who is subject to frequent epilepsy, being perfectly cognizant of the surrounding objects, and a person of considerable judgment, is placed among the most intellectual class.

To assemble all the imbecile, ruminative, and cogitabund patients in one apartment, would, on the one hand, greatly increase the difficulty and labour of keeping them and their habitation clean and comfortable; and, on the other, would throw too many of the irascible within the sphere of dangerous collision. When there are only eight rooms for seventy patients, it may well be supposed that each must contain, upon any principle of association, cases widely dissimilar.

After all, is it certain that the indiscriminate mixture of various cases of mania is an evil? May not the contrast, the recoil of the feelings in maniacs of opposite feelings, on being made to live together, produce a salutary effect, by powerfully dispelling the illusions of reverie,—by impressing on the patient's mind the appalling conviction that he is deemed a fit associate for the wretched objects, whose follies are so unlike his own,—and thus by throwing the mind back upon what remains of common sense? Could we suppose an assemblage of insane patients, all nearly of the same tempers, and nearly of the same type and degree of folly, their social relations being pretty much in harmony with their cogitations, would probably fortify and substantiate their illusions.

Differences of temper are, however, of course considered as of practical importance, and, whatever advantages could be gained from classification, are gained by prompt attention to discipline. The violent are not dangerous to the timid, for the peccant individual is immediately seized, and is either confined by himself in a small room reserved for this purpose, or, being allowed to take exercise in his strait-waistcoat, his example may be salutary to such as are capable of reflection.

One essential motive of classification is always attended to: the insane sick are placed in a room together.

Classification according to temper is adhered to as much as possible with the officers: the two dormitories are apportioned with a view to this, and a small room, attached to the keeper's dwelling, is made to accommodate two convalescent officers. No admixture of the officers with the soldier patients at present exists, although until lately this has, in certain cases, been deemed admissible by my predecessors and by myself. Thus, one of these cases was an instance of complete mental prostration, where all sense of decency in the time and place of obeying the calls of nature had long since disappeared. Another, nearly similar, was complicated with paralysis. In the third, the moral sense alone had fled, and an incorrigible propensity to stealing, to insolence, and violence, rendered this subject an alien to the feelings of his station, and a nuisance to his equals in rank. Views of expediency and of comfort were listened to in placing among the privates these three cases, where the dissolution of all artificial distinctions had brought the two ranks of society to the same common level of degradation. Their diet was sent to them from the officer's mess. Such arrangement, however, being considered irregular in its principle, was for the future prohibited.

Patients are admitted into the asylum by the authority of the secretary at war, or commander in chief, or director general of the medical department. Upon all patients, of whatever rank, a board of medical officers is held as soon as possible after admission, to ascertain the medical propriety of that measure. The same sanctions are required for the discharge of patients; but it will be readily anticipated that the latter measure is one of much more difficult decision than the former. Surrounded by the quiet of an asylum, the phrenzy of the violent often remains quiescent, to explode afresh on the re-excitement of society; or the orgies which had, perhaps, proved the source of that phrenzy, though suspended, have left their clinging hold upon the habits of the emancipated convalescent, to drag him back to his cell, through scenes of violence and peril. Again, the timid, retiring melancholic leans with the confidence of convalescence upon the protection of the walls of our asylum. Turned out upon the world, though perfectly sane, his inexperience, his indolence, his want of robust confidence in his own powers, subject him to chicanery and deceit; and these, in turn, or the apprehension of them, to relapse. Yet, in either case, the longer detention of a person in his sane mind is an evil: it may be dangerous, by inducing feelings of despair and suicidal gloom; and, at any rate, it may be mischievous, by weakening his mind by protracted tutelage, and deprivation of experience. To solve this dilemma requires a co-operation on the part of the friends and relatives, which is not always readily extended, or compatible with their means, or there may be no relatives surviving. Prudential calculations thus sometimes traverse our medical views; an observation very trite, indeed, but so much the more interesting to those to whom it is suggested, by its foundation in a frequent source of embarrassment. The instructions of the Army Medical Board are always afforded to decide every difficulty, where a sound decision is practicable.

As the disease has always existed some time before the admission of the patient, the cause is often obscure. We have never seen it in operation; and its complication with hereditary, or other predisposing causes, is necessarily an obscure inquiry.

The different causes of derangement, *assigned* in such of the cases as have received any illustration of this kind, are here enumerated.

| Physical Causes. | | Moral Causes. | | Hereditary. |
|---|---|---|---|---|
| Fever | 6 | Religious fanaticism | 6 | 12 |
| Wound on head | 5 | Love | 2 | |
| Malformation of head | 2 | Family misfortunes | 3 | |
| Coup de soleil | 1 | Fright (in females) | 2 | |
| Drinking | 3 | Disappointment | 1 | |
| Apoplexy | 1 | Violent passion (rage?) | 1 | |
| Epilepsy | 5 | Remorse | 1 | |

In attempting to assign their respective influence to each division of causes, the moral and the physical, I shall briefly illustrate the different kinds of cases which are to be found in the asylum.

The *moral* causes appear, at first sight, least adequate to have produced singly their attributed effect. Sudden calamity, ardent or depressing emotions, as love, remorse, religious fanaticism, may change or deteriorate the character,—may make a man vindictive and cruel, or timid and irresolute, or pensive and prone to absorption or idle reverie; but the powerful and rousing correctives of the rude jostling of mankind, and the spur of necessity—the pains of hunger, appear generally sufficient to dispel the most cherished illusions suggested to a previously sound mind. The relations which should connect these illusions with the surrounding objects, to invest them with reality, and to obtain for them belief in the patient's mind,—these relations are not supplied by the mere intensity of the image. An enthusiast may, in idea, fancy himself a king,—may form to himself a conception of that office as enjoyed by him; but will the mere vividity of the impression make him see, in the beggarly furniture of his garret, the gorgeous accompaniments of regal splendour? We are told by authors that it may; but no analogy seems to lie between the strongest notional conceptions of a sane fancy and the belief in their reality, which, in the insane mind, marks the perversion of the judgment and of the senses. There is in mania something organically wrong, which, not being ostensibly connected with the operation of unassisted moral causes, is not accounted for by their alleged application.

Reverie and absence of mind, intense absorption, trains of associated images, day-dreams, or brown study, do, however, constitute one species of unsoundness of mind, and unfit the patient very thoroughly for the usual business of life. Yet that state differs only in degree from the usual and healthful exercise of the imagination in poets, and perhaps in painters. The morbid habit is only stronger than the healthful; belief in the real presence is not experienced: rouse the dreamer from his visions, his confused start and abashed expression mark his consciousness of the surrounding actual circumstances, and prove that he had merely laid the reins loosely upon the neck of his imagination. Morbid reverie is probably produced by moral causes, by pleasurable or painful emotion, including among the latter ennui; and it constitutes the characteristic type of several cases in Fort Clarence, both among the officers and soldiers. It is a state which is apt to grow upon convalescents during the lucid interval, during the tedium and dreary indolence of confinement. Of these patients in Fort Clarence, some are visited by cheerful musings, some by dull sombrous suggestions; and these cases again may be subdivided by as many individual peculiarities as there are peculiar temperaments.

When belief in extravagant absurdity (or delirium) begins, reverie ends; the impression acquires fixity. The state of a patient in Fort Clarence, who believes that a young man, of the name of Livingston, has got into his flank in the shape of a cock, is widely different from that I have described under the former class: his aberrations arose from a wound of the head. An officer, who had lived a very free life, believed himself surrounded by phantoms of his relatives in miniature, who hissed at him, spit at him, and denounced eternal damnation. He could reason about the justice of such retribution, compared with his venial crimes, but could not argue down the impression or the belief. His hallucination yielded at last to the power of time, quiet, and regularity, rather (as I believe) than to medicines; and he lately departed cured. Many others see, or have seen, angels, saints, &c.; and all those whom I have met with had been free drinkers, or had suffered from some physical lesion, from fever, insolation, wound, or their cases were complicated by epilepsy or other structural derangement of the cerebral organs, or by functional or sympathetic disease, from affections of the gastric or hepatic organs. These causes, by producing cerebral plethora, may well be supposed to have deranged the finer texture of those organs of sense, through which the mind is rendered cognizant of external objects. Judgment, or the perception of the relations of these objects, is dimmed by the false medium.

One species of madness there is which may be produced by moral causes; it is that which is characterised, not by diseased perception, but by mere thirst after violent and extravagant excitement or mental succussion,—a craving after strong sensation, ungovernable passion. It may be considered as a convulsion of the mind; and, as that disease in the body may be excited by tooth-ache or other pain, so the mental pains, much more intolerable, seek relief in substituted horror, by which a change in the kind and in the degree of suffering is produced. One of the most interesting cases of this kind was attributed to love: the disease was periodical, the patient frantic, outrageous in alternate mirth and ferocity. Whether it wore itself out, or was really checked by the numerous and severe remedies, a complete cure was obtained, and he emerged into robust sanity of mind. [illegible]

It remains to notice in this place the large class of idiots, congenital, hereditary, or converted, from the acute stage of mania to this, the chronic stage; and it must be added, that, in many of the old cases, a mixture of all the types of alienation is seen, constituting *chaotic* madness. The enumeration of the different types of mental disease observed at Fort Clarence, is thus completed.

Dissection of the fatal cases has not thrown any satisfactory light on the pathology of mania. In many instances the most minute examination could detect no organic lesion; in others, marks of vascular congestion were found, and plethora of the cerebral vessels. Effusions of serum, in morbid quantity, were found in some cases of idiotism, where such appearances might have been anticipated. Roughness and absorption of the posterior clinoid processes were frequently, but not universally, found.

The mode of treatment adopted in Fort Clarence is not peculiar to the place; neither is the disappointment, which has so often followed every curative effort. The distribution of these into physical and moral means, is implied by the nature of the respective causes. That the powers of either plan of treatment are often considerable in recent cases, we have had opportunities of verifying.

The physical cure, where maniacal excitement is present, is sought, 1st, by diminishing the afflux of blood to the head, either by general or local abstraction of blood; or, 2d, by the superinduction of some other derivative evacuation, as by purging or vomiting: the emetic preferred is the sulphat of copper; the cathartics vary according to occasional circumstances. The oil of turpentine, in doses of half an ounce to six drachms, has, in several cases, appeared decidedly serviceable; elaterium, gamboge, oil of croton, &c. &c. 3d. By setting up some vascular action or secretion, derivative of the morbid excitement, from its pernicious concentration upon the sensorial organs, as by blistering, setons, nettles externally applied, mercurial excitement carried the length of gentle salivation,—warm bath; or, 4th, by suspending the nervous sensibility, and inducing quiescence of all the functions, by narcotics and sedatives,—cold. The narcotics are stramonium, prussic acid, and opium. Cold is applied by affusion from a great height on the head, while the body is immersed in the warm bath. In this last way, the circular swing, although in its first effect decidedly an emetic, was, by its early introducers, believed to act. During the collapse and distressing sickness and languor produced by this dreaded instrument, penitence and strenuous promises of amendment have sometimes been proffered; they were forgotten as soon as the sickness went off.

What is called the moral treatment comprehends discipline, and every thing connected with mental training. The exercise and amusements of the patients are, by their effects in changing the trains of thought, and weaning the patient from his reveries, reducible to the same head. These resources are with us very limited. To find the means of exertion, varied according to the tastes and education of individuals, or even of groups of individuals, (as by the establishment of any easily-learnt trade,) would require an appropriation of capital much beyond the funds accruing from the stoppages of the patients. The only available means seemed to be the conversion of the grounds of the 'fort into an extensive kitchen-garden, to be cultivated by the patients, by which means economy and healthful labour, it was hoped, might be jointly ensured. On trial, however, the soil was found to be so very thin and barren, as to render all attempts at extensive culture chimerical, without creating a new mould by manure; consequently, by much outlay. A stripe along the better part is, however, at present under the spade of the maniacs, and some good must come of the attempt.

The soldiers of Britain cultivate few accomplishments, by which to embellish their retirement. While the less complete subdivision and appropriation of labour in foreign countries has left to their peasantry, and consequently to their soldiers, a comparatively large share of versatile ingenuity and variety of resources, (as we have seen in the neat productions of the French prisoners,) the concentration of the powers of the Englishman to the few automatic, unintelligent motions, by which, as a small part of an immense machine, he co-operates to the production of a great effect, increases his peculiar utility, but impairs his general powers of invention. Hence none of those neat models, no dice, no chess-men, no cribbage-boxes, nor straw hats, issue from the convalescents of English hospitals.

One patient alone in Fort Clarence, however, is a votary of the fine arts, and his paintings are exceedingly original, quaint, and ingenious. His pencil, a tuft of the down of the thistle, tied on a small stick plucked out of the birch-brooms, (which he seems to prefer to the pencils of the shops, which have been supplied him,) is always dedicated to the higher walks of the art,—battle pieces, allegories, processions, and ideal portraits

4

of persons, living characters, whom he has never seen. It is interesting to contemplate the products of mere genius, unassisted by the precepts of the art. With him must be associated the clarionet-player, who once belonged to a military band, and an occasional amateur taylor: upon the rest the *muses* have never smiled.

The moral remedies for reverie are mental industry, reading, and difficult study,—above all, the practice of the world, where it can be had with safety. In Fort Clarence the studies of this class are carefully encouraged, when any portion of capacity remains, or has ever been cultivated. Lieutenant H—— and Hospital-mate S—— are allured to study by placing classical authors in their way. They are both promenaded into the town every day, under the wardship of the keeper. Ensign O—— was made to work an arithmetical account, connected with the hospital, every day.

No delusive coaxing or flattery of the absurdities of the patients is practised, except for the occasional purpose of ascertaining the particular bent or type of their maladies. Such of them as are, in any degree, amenable to the laws of common reason, are treated very much in the same way as if they were sane people: they are made to feel the yoke of necessity. Reasoning is seldom attempted, except where a ray of sense appears to have broke in upon their fatuity; nor any particular delicate mental management, by setting up opposite prejudices; the period when, if ever, the latter mode of treatment could succeed, is past long before the admission of the patients. Besides, except in the mere case of reverie, there is depravity of judgment; the patient sees through a false medium. To correct his wrong impression, were it possible, could only be done at the expence of setting up another in its stead. All that could be hoped from subtlety of psychological treatment, is here sought from the quiet of the place, from the confinement, the regular hours, exercise, the cold severity of deportment of the attendants towards the froward, the friendship and protection extended towards the timid and amiable. The sallies of the ferocious are checked by mere coercion, if that type is attended with complete prostration of the perceptive and discriminative powers: if these remain in part, if the case is one of mere ardour and disposition to violence and extravagance, from the sudden rising of some internal painful feeling, moral or physical, punishment is awarded, in conjunction with such physical remedies as may appear indicated; and correction is often attended with very striking temporary advantage. It is inflicted by means of the circular swing, or by solitary confinement. The former of these means is seldom used for any other purpose than punishment, as I imagine that a remedial power, which, by the mode of its application, implies an harsh and violent infliction, must labour under the drawback of appearing to the patient a gratuitous act of oppression and cruelty.

Such is the moral treatment, if such it can be called, of insanity, as practised in Fort Clarence.

On the proportion of cures I have no other observation to make, except that I hope it will be found to be not inferior to the number of cures achieved in other establishments; regard being had to the comparative length of time during which the disease may have lasted previous to admission.

The deaths have all proceeded from corporeal ailment, unconnected with the insane malady, or, at least, only remotely and indirectly so connected. They have proeeeded chiefly from tubercular consumption, to which melancholic and imbecile patients are peculiarly liable. Some, from vociferation proving an exciting cause of tubercular disease; some, from occasional exposure of the body (neck and breast,) to cold; but most from that torpor and debility which is the attendant upon mental absorption. In that languid, lethargic state, the latent seeds of disease are speedily developed; temperament is rendered morbid, and predisposition is rendered active. Consumption is also occasionally produced by gastric and hepatic derangement, in the patients who arrive from hot climates. Some have died of paralysis, and one from phrenitis acuta.

Such is the condition of the patients in Fort Clarence; an institution which must be interesting to those whose philanthropy is able to subdue the natural aversion excited by the dreary, disgusting spectacle of man in his most degraded state. One grand desideratum is apparent in every part, the want of room for classification: it will speedily be wanting for comfort also, unless some addition is made by building.

